# Editorial: Sarcoidosis—The great mimicker

**DOI:** 10.3389/fmed.2022.990714

**Published:** 2022-08-02

**Authors:** Peter Korsten, Mehdi Mirsaeidi, Nadera J. Sweiss

**Affiliations:** ^1^Department of Nephrology and Rheumatology, University Medical Center Göttingen, Göttingen, Germany; ^2^Division of Pulmonary, Critical Care, and Sleep Disease, College of Medicine-Jacksonville, University of Florida, Jacksonville, FL, United States; ^3^Division of Rheumatology, Department of Medicine, University of Illinois at Chicago, Chicago, IL, United States

**Keywords:** sarcoidosis, chronic granulomatous disease, pulmonary sarcoidosis, hypercalcemia, case reports

## Introduction

Sarcoidosis has been and remains a challenge for patients and physicians alike. Even in the year 2022, the pathophysiology of sarcoidosis is still incompletely understood. With this Research Topic entitled “Sarcoidosis—The Great Mimicker,” we aimed to shed light on the various aspects of this enigmatic disease. We are thankful and indebted to all authors, reviewers, and external editors who have contributed with their research papers and time to make this a thriving collection of articles. We received many submissions indicating that sarcoidosis attracts researchers from various backgrounds and specialties. With this editorial, we will give an overview of the topics covered in the Research Topic and place them in the context of the current research landscape.

## Learning from case reports

**Figure 1:**
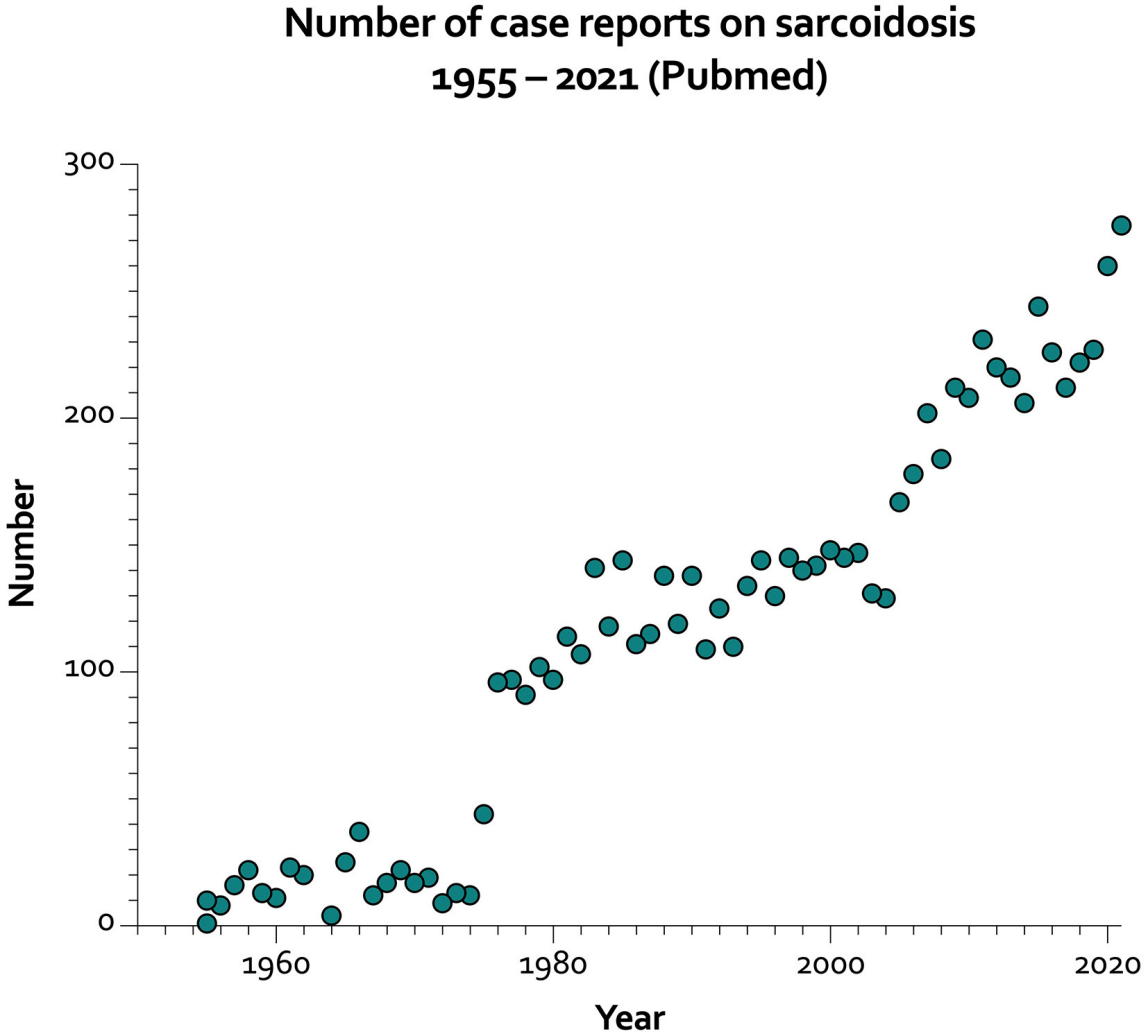
Number of case reports on sarcoidosis per year from 1955 to 2021 (https://pubmed.ncbi.nlm.nih.gov/; last accessed July 8th, 2022).

The search terms (Sarcoidosis[Title]) AND (Case Reports[Filter]) in the commonly used database Pubmed retrieved a steadily increasing number of cases over the years and a total number of 7.280 reports. Starting in 1955, only one case was reported, which increased to 276 as of 2021 (Figure 1). Thus, case reports of sarcoidosis or its mimickers are a valuable educational tool to capture the frequent and infrequent manifestations of the disease. In this regard, two case reports were accepted for this collection of articles. The first article, written by Tirelli et al. describes a case of concurrent adenocarcinoma of the lung and coexistent sarcoidosis, which is rare and therapeutically challenging. A recent systematic review identified nine case reports and highlighted that meticulous diagnostic testing is essential to differentiate metastatic disease from sarcoid or sarcoid-like lesions (1).

The following case report by De Cinque et al. focuses on testicular sarcoidosis, an exceedingly rare manifestation of sarcoidosis. They reviewed the literature and identified, including their case, only 20 publications where testicular sarcoidosis was the primary manifestation of sarcoidosis. Most interestingly, they described contrast-enhanced ultrasound (CEUS) features, which can be helpful in discerning sarcoidosis from other malignant testicular lesions. Specifically, sarcoid lesions are hypoechoic and show hypo-enhancement on CEUS, a clinically relevant point.

Lastly, Manansala et al. describe a case series of COVID-19 in sarcoidosis patients of African American ethnicity. They report on five cases; one ultimately expired due to a thromboembolic event. This report, published relatively early in the pandemic, was reassuring for patients with sarcoidosis, especially African American patients, who usually suffer more from sarcoidosis than other ethnicities (2, 3). However, a single-center study, which analyzed patients seen from July to December 2020, suggested that the hospitalization rates and the mortality from COVID-19 in sarcoidosis may be increased (4).

## Reviews on cancer, novel treatments, pitfalls, and the microbiome

In the first of four review papers in this Research Topic, El Jammal et al. review the relationship between sarcoidosis and cancer, which complements the case report by Tirell et al. mentioned above. The authors summarize the available literature on sarcoidosis and its relationship to cancer; the main concern is the occurrence or co-occurrence of lymphomas. Further, they highlight the importance of a dedicated histopathologic analysis because some malignancies with granulomatous features may otherwise be missed. In addition, it is essential to consider infectious complications in patients with a known cancer diagnosis, especially opportunistic infections.

Next, Boleto et al. provided an overview of novel therapeutic targets for treating refractory sarcoidosis. They focused on non-tumor necrosis factor-alpha inhibitor monoclonal antibodies. Unfortunately, the available clinical trial data for many agents, including interleukin (IL)-17 and−12/23-antagonists or B-cell modulating agents, were negative. Clinical trials for anti-IL-6, anti-IL-1, and Janus kinase inhibitors are ongoing and have not been published yet. In light of the recently published American Thoracic Society/European Respiratory Society recommendations for the treatment of sarcoidosis, which confirmed the lack of robust evidence derived from high-quality clinical trials, these trial results are eagerly awaited (5). A recent, very detailed report highlighted the beneficial role of tofacitinib in sarcoidosis (6) after the first very encouraging reports for cutaneous sarcoidosis (7).

Adding to the review papers on dedicated topics concerning sarcoidosis is the article by Narula and Iannuzzi on pitfalls and challenging mimickers. Here, the authors reviewed the most common infections and non-infectious diseases across frequent (such as pulmonary sarcoidosis) and infrequent (neurosarcoidosis) manifestations. In addition, they highlight diseases and conditions that treating physicians always need to be aware of, such as drug-induced sarcoid reactions, common-variable immunodeficiency with granulomas, and differential diagnoses for hypercalcemic syndromes.

The last review of the topic by Chioma et al. described the role of the lung microbiome in interstitial lung diseases (ILD), including sarcoidosis. Among the few dedicated studies available, the evidence for alterations in the gut or lung microbiome is mixed. In sarcoidosis specifically, alterations of the microbiome in the lungs have not been firmly established.

## Original papers

A total of four original papers were included in the topic. The first paper by Cacciatore et al. analyzed a French cohort regarding acute vs. chronic sarcoid arthropathy. In short, chronic arthropathy tended to be less symmetric, had more wrist involvement, and more often required second- or third-line therapies.

The subsequent exciting investigation by Cameli et al. identified serum and urinary calcium levels as a useful biomarker in sarcoidosis. The investigators compared sarcoidosis patients with idiopathic pulmonary fibrosis and hypersensitivity pneumonitis. Further, receiver operating characteristics curve analysis revealed an excellent specificity (89.7%) of hypercalciuria for sarcoidosis vs. non-sarcoidosis ILD and a specificity of 82.5% for fibrotic sarcoidosis vs. non-sarcoidosis ILD. This paper highlights the importance of calcium and vitamin D dysregulation in sarcoidosis, which is an area deserving of further investigation.

The following paper by Nickles et al. analyzed similarities between discoid lupus erythematosus (DLE), cutaneous sarcoidosis (CS), and psoriasis by gene co-expression networks. These complex analyses revealed seven common hub genes in DLE and CS: TLR1, ITGAL, TNFRSF1B, CD86, SPI1, BTK, and IL10RA. Furthermore, these analyses also identified several disease-type specific hub genes. These results identified potential molecular targets for treating skin lesions of these diseases.

Lastly, Vagts et al. used unsupervised clustering in a cohort of sarcoidosis patients who had undergone a positron emission tomography (PET) scan as part of their workup. A detailed analysis of clinical, laboratory, and imaging features revealed three clusters (African Americans with quiescent disease, African Americans with chronically active and more frequent extrathoracic disease, and Caucasians with more acute illness). In addition, the data showed a reduction in lymphocyte counts in cluster 3 and high PET avidity in clusters 2 and 3.

These results are relevant since the current recommendations for the diagnosis and detection of sarcoidosis (8) still very much rely on the exclusion of similar disorders. With further research, one goal should be to add confidence in the diagnostic certainty when sarcoidosis is suspected.

## Conclusions

Sarcoidosis remains an enigmatic and complicated disease. Within this Research Topic, experts in the field have shared their insights, experience, and research data to deepen our understanding of sarcoidosis. In our view, the agenda for future research includes but is not limited to: (1) what does assist us in making a confident diagnosis of sarcoidosis?, (2) the development of disease clustering criteria for clinical trials, (3) a definition of an applicable response index for measuring outcomes in clinical trials, (4) further explore the role of calcium/vitamin D metabolism in sarcoidosis, and (5) investigate the molecular mechanisms underlying the pathogenesis of the disease. Specialists from all fields, such as pulmonologists, rheumatologists, dermatologists, and neurologists, among many others, should embrace these challenges to improve the diagnosis and treatment of sarcoidosis patients for whom very few therapeutic options are available.

## Author contributions

PK conceived the article, wrote the manuscript, and created the figure. NS and MM reviewed and edited the paper. All authors contributed to the article and approved the submitted version.

## Conflict of interest

Author PK has received honoraria or travel support from Abbvie, Amgen, AstraZeneca, Boehringer Ingelheim, Bristol-Myers-Squibb, Chugai, Galapagos Biopharma, GlaxoSmithKline, Janssen-Cilag, Lilly, Pfizer, and Sanofi-Aventis, all unrelated to this paper. Author PK received research grants from GlaxoSmithKline and Diamed Medizintechnik GmbH, all unrelated to this paper. Author MM reports research awards from Mallinckrodt, advisory membership in Mallinckrodt. The remaining author declares that the research was conducted in the absence of any commercial or financial relationships that could be construed as a potential conflict of interest.

## Publisher's note

All claims expressed in this article are solely those of the authors and do not necessarily represent those of their affiliated organizations, or those of the publisher, the editors and the reviewers. Any product that may be evaluated in this article, or claim that may be made by its manufacturer, is not guaranteed or endorsed by the publisher.

## References

[1] Srinivasan M Thangaraj SR Arzoun H Govindasamy Kulandaisamy LB Mohammed L. The association of lung cancer and sarcoidosis: a systematic review. Cureus. (2022) 14:e21169. 10.7759/cureus.21169 35103216 PMC8776520

[2] Hena KM. Sarcoidosis epidemiology: race matters. Front Immunol. (2020) 11:537382. 10.3389/fimmu.2020.537382 33042137 PMC7522309

[3] Gerke AK Judson MA Cozier YC Culver DA Koth LL. Disease burden and variability in sarcoidosis. Ann Am Thorac Soc. (2017) 14:S421–8. 10.1513/AnnalsATS.201707-564OT 29087725 PMC5802572

[4] Baughman RP Lower EE. COVID-19 infections in sarcoidosis: a prospective single center study of 886 sarcoidosis patients. Sarcoidosis Vasc Diffuse Lung Dis. (2021) 38:e2021029. 10.36141/svdld.v38i2.11646 34316261 PMC8288208

[5] Baughman RP Valeyre D Korsten P Mathioudakis AG Wuyts WA Wells A. ERS clinical practice guidelines on treatment of sarcoidosis. Eur Respir J. (2021) 2004079. 10.1183/13993003.04079-2020 34140301

[6] Damsky W Wang A Kim DJ Young BD Singh K Murphy MJ. Inhibition of type 1 immunity with tofacitinib is associated with marked improvement in longstanding sarcoidosis. Nat Commun. (2022) 13:3140. 10.1038/s41467-022-30615-x 35668129 PMC9170782

[7] Damsky W Thakral D Emeagwali N Galan A King B. Tofacitinib treatment and molecular analysis of cutaneous sarcoidosis. N Engl J Med. (2018) 379:2540–6. 10.1056/NEJMoa1805958 30586518 PMC6351852

[8] Crouser ED Maier LA Wilson KC Bonham CA Morgenthau AS Patterson KC. Diagnosis and detection of sarcoidosis. An official american thoracic society clinical practice guideline. Am J Respir Crit Care Med. (2020) 201:e26–51. 10.1164/rccm.202002-0251ST 32293205 PMC7159433

